# 3,4-Di­hydro-1*H*-benzo[*c*]chromene-1,6(2*H*)-dione

**DOI:** 10.1107/S1600536813010210

**Published:** 2013-04-20

**Authors:** Liang-Yan Cui, Yan He, Xue-Sen Fan

**Affiliations:** a School of Chemistry and Chemical Engineering, Henan Normal University, Henan 453007, People’s Republic of China

## Abstract

In the title compound, C_13_H_10_O_3_, the pyran­one and benzene rings are almost coplanar, making a dihedral angle of 1.9 (1)°. The cyclo­hexenone ring adopts an envelope conformation, with a methyl­ene C atom located at the flap and displaced by 0.639 (3) Å from the mean plane of the other five atoms. In the crystal, pairs of weak C—H⋯π inter­actions occur between inversion-related mol­ecules.

## Related literature

For applications of benzo[*c*]chromen-6-ones, see: Schmidt *et al.* (2003 ▶); Pandey *et al.* (2004 ▶); Matsumoto & Hanawalt (2000 ▶); Sun *et al.* (2006 ▶). For the synthesis, see: Fan *et al.* (2012 ▶).
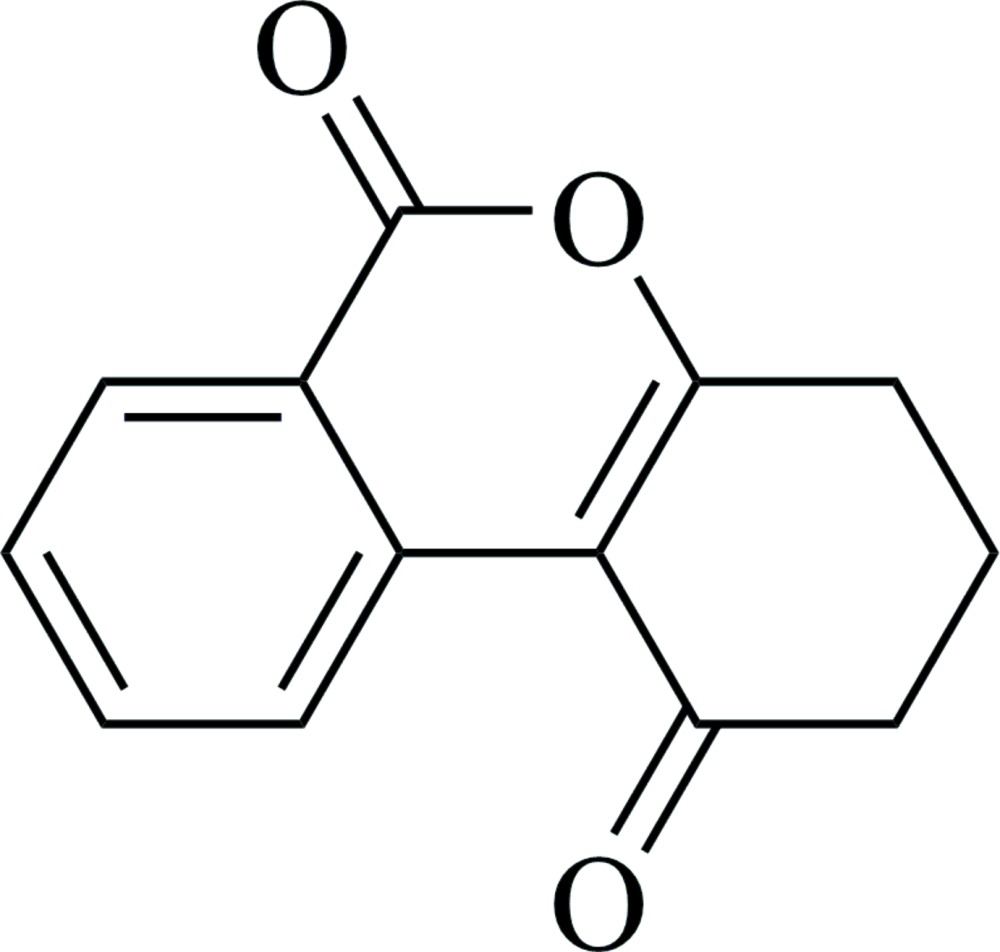


## Experimental

### 

#### Crystal data

C_13_H_10_O_3_
*M* *_r_* = 214.21
Monoclinic, 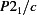

*a* = 8.234 (3) Å
*b* = 10.199 (3) Å
*c* = 11.927 (4) Å
β = 97.439 (4)°
*V* = 993.1 (6) Å^3^
*Z* = 4
Mo *K*α radiation
μ = 0.10 mm^−1^
*T* = 296 K
0.47 × 0.41 × 0.31 mm

#### Data collection

Bruker SMART 1000 CCD area-detector diffractometer
6998 measured reflections
1843 independent reflections
1517 reflections with *I* > 2σ(*I*)
*R*_int_ = 0.027

#### Refinement

*R*[*F* ^2^ > 2σ(*F* ^2^)] = 0.077
*wR*(*F* ^2^) = 0.229
*S* = 1.18
1843 reflections
145 parameters
H-atom parameters constrained
Δρ_max_ = 0.33 e Å^−3^
Δρ_min_ = −0.29 e Å^−3^

### 

Data collection: *SMART* (Bruker, 2007 ▶); cell refinement: *SAINT* (Bruker, 2007 ▶); data reduction: *SAINT*; program(s) used to solve structure: *SHELXTL* (Sheldrick, 2008 ▶); program(s) used to refine structure: *SHELXTL*; molecular graphics: *SHELXTL*; software used to prepare material for publication: *SHELXTL*.

## Supplementary Material

Crystal structure: contains datablock(s) I, global. DOI: 10.1107/S1600536813010210/xu5693sup1.cif

Structure factors: contains datablock(s) I. DOI: 10.1107/S1600536813010210/xu5693Isup2.hkl

Supplementary material file. DOI: 10.1107/S1600536813010210/xu5693Isup3.cdx

Supplementary material file. DOI: 10.1107/S1600536813010210/xu5693Isup4.cml

Additional supplementary materials:  crystallographic information; 3D view; checkCIF report

## Figures and Tables

**Table 1:** Hydrogen-bond geometry (Å, °) *Cg* is the centroid of the C1–C6 benzene ring.

*D*—H⋯*A*	*D*—H	H⋯*A*	*D*⋯*A*	*D*—H⋯*A*
C11—H11*B*⋯*Cg* ^i^	0.97	2.91	3.723 (5)	142

Symmetry code: (i) 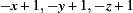.

**Fig. 1.**
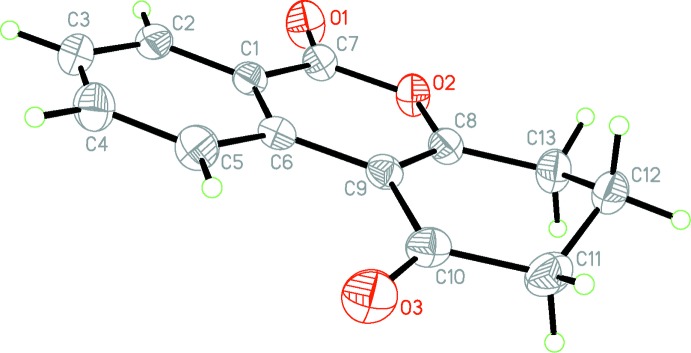
Molecular structure of the title compound, with displacement ellipsoids drawn at the 30% probability level.

**Fig. 2.**
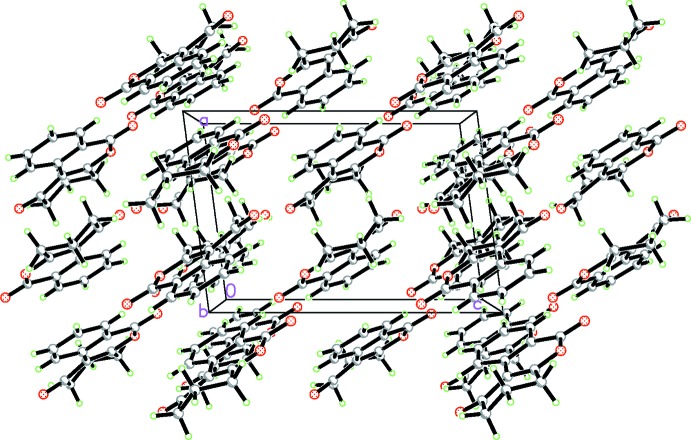
Crystal structure of the title compound with view along the b axis.

## supplementary crystallographic information

### Comment

Benzo[*c*]chromen-6-ones constitute one of the major classes of pharmacologically relevant natural products and display a wide range of biological activities (Schmidt *et al.*, 2003; Pandey *et al.*, 2004; Matsumoto *et al.*, 2000; Sun *et al.*, 2006). As part of our research (Fan *et al.* 2012), we have synthesized the title compound (I), and report its crystal structure here.

The title compound, C_13_H_10_O_3_, consists of three fused six-membered rings, benzene, pyranone and cyclohexenone ring. The pyranone ring is in the middle. All the bond lengths and bond angles are within normal ranges. The pyranone ring and the benzene ring are almost coplanar with a dihedral angle of 1.9 (1) °. The cyclohexenone ring adopts an envelope conformation, a methylene C atom located on the flap and displaced from the mean plane of the other five ring atoms (C8—C11/C13) by 0.639 (3) Å.

In the crystal, weak intermolecular C—H···π interactions occur between benzene ring and methylene group of adjacent molecules, the separation between the centroid of benzene ring and methylene H atom being 2.91 Å.

### Experimental

The title compound was synthesized following the previously reported procedure (Fan *et al.*, 2012). Single crystals, suitable for X-ray diffraction analysis, were obtained by slow evaporation of the solvents from a petroleum ether-ethyl acetate (5:1 *v*/*v*) solution of the title compound.

### Refinement

H atoms were positioned geometrically and refined using riding model with C—H = 0.93–0.97 Å and *U*_iso_(H) = 1.2*U*_eq_(C).

### Crystal data

**Table:**

C_13_H_10_O_3_	*F*(000) = 448
*M**_r_* = 214.21	*D*_x_ = 1.433 Mg m^−^^3^
Monoclinic, *P*2_1_/*c*	Mo *K*α radiation, λ = 0.71073 Å
Hall symbol: -P 2ybc	Cell parameters from 2699 reflections
*a* = 8.234 (3) Å	θ = 2.5–27.2°
*b* = 10.199 (3) Å	µ = 0.10 mm^−^^1^
*c* = 11.927 (4) Å	*T* = 296 K
β = 97.439 (4)°	Block, colorless
*V* = 993.1 (6) Å^3^	0.47 × 0.41 × 0.31 mm
*Z* = 4	

### Data collection

**Table:**

Bruker SMART 1000 CCD area-detector diffractometer	1517 reflections with *I* > 2σ(*I*)
Radiation source: fine-focus sealed tube	*R*_int_ = 0.027
Graphite monochromator	θ_max_ = 25.5°, θ_min_ = 2.5°
φ and ω scans	*h* = −9→9
6998 measured reflections	*k* = −12→12
1843 independent reflections	*l* = −14→14

### Refinement

**Table:**

Refinement on *F*^2^	Primary atom site location: structure-invariant direct methods
Least-squares matrix: full	Secondary atom site location: difference Fourier map
*R*[*F*^2^ > 2σ(*F*^2^)] = 0.077	Hydrogen site location: inferred from neighbouring sites
*wR*(*F*^2^) = 0.229	H-atom parameters constrained
*S* = 1.18	*w* = 1/[σ^2^(*F*_o_^2^) + (0.0741*P*)^2^ + 2.0297*P*] where *P* = (*F*_o_^2^ + 2*F*_c_^2^)/3
1843 reflections	(Δ/σ)_max_ < 0.001
145 parameters	Δρ_max_ = 0.33 e Å^−^^3^
0 restraints	Δρ_min_ = −0.29 e Å^−^^3^

### Special details

**Table:**

Geometry. All e.s.d.'s (except the e.s.d. in the dihedral angle between two l.s. planes) are estimated using the full covariance matrix. The cell e.s.d.'s are taken into account individually in the estimation of e.s.d.'s in distances, angles and torsion angles; correlations between e.s.d.'s in cell parameters are only used when they are defined by crystal symmetry. An approximate (isotropic) treatment of cell e.s.d.'s is used for estimating e.s.d.'s involving l.s. planes.
Refinement. Refinement of *F*^2^ against ALL reflections. The weighted *R*-factor *wR* and goodness of fit *S* are based on *F*^2^, conventional *R*-factors *R* are based on *F*, with *F* set to zero for negative *F*^2^. The threshold expression of *F*^2^ > σ(*F*^2^) is used only for calculating *R*-factors(gt) *etc*. and is not relevant to the choice of reflections for refinement. *R*-factors based on *F*^2^ are statistically about twice as large as those based on *F*, and *R*- factors based on ALL data will be even larger.

### Fractional atomic coordinates and isotropic or equivalent isotropic displacement parameters (Å^2^)

**Table:**

	*x*	*y*	*z*	*U*_iso_*/*U*_eq_	
C1	0.1515 (4)	0.4254 (4)	0.4108 (3)	0.0372 (8)	
C2	0.0900 (5)	0.2983 (4)	0.3970 (4)	0.0462 (10)	
H2	0.0294	0.2737	0.3290	0.055*	
C3	0.1189 (5)	0.2095 (4)	0.4836 (4)	0.0556 (11)	
H3	0.0797	0.1242	0.4741	0.067*	
C4	0.2063 (6)	0.2472 (5)	0.5847 (4)	0.0577 (12)	
H4	0.2239	0.1871	0.6437	0.069*	
C5	0.2681 (5)	0.3725 (4)	0.6002 (3)	0.0494 (10)	
H5	0.3266	0.3958	0.6693	0.059*	
C6	0.2435 (4)	0.4652 (4)	0.5127 (3)	0.0361 (8)	
C7	0.1173 (5)	0.5166 (4)	0.3171 (3)	0.0409 (9)	
C8	0.2628 (4)	0.6829 (4)	0.4336 (3)	0.0388 (9)	
C9	0.3044 (4)	0.6001 (4)	0.5209 (3)	0.0364 (8)	
C10	0.4169 (5)	0.6504 (4)	0.6187 (3)	0.0448 (10)	
C11	0.4572 (6)	0.7945 (5)	0.6214 (4)	0.0575 (12)	
H11A	0.4649	0.8249	0.6990	0.069*	
H11B	0.5640	0.8061	0.5967	0.069*	
C12	0.3362 (5)	0.8789 (4)	0.5496 (4)	0.0522 (11)	
H12A	0.3787	0.9675	0.5474	0.063*	
H12B	0.2345	0.8823	0.5824	0.063*	
C13	0.3036 (6)	0.8247 (4)	0.4302 (4)	0.0517 (11)	
H13A	0.2133	0.8719	0.3880	0.062*	
H13B	0.3998	0.8366	0.3922	0.062*	
O1	0.0424 (4)	0.4948 (3)	0.2258 (2)	0.0618 (9)	
O2	0.1742 (3)	0.6438 (3)	0.3351 (2)	0.0457 (7)	
O3	0.4831 (4)	0.5785 (3)	0.6928 (2)	0.0663 (10)	

### Atomic displacement parameters (Å^2^)

**Table:**

	*U*^11^	*U*^22^	*U*^33^	*U*^12^	*U*^13^	*U*^23^
C1	0.0344 (18)	0.038 (2)	0.0388 (19)	0.0033 (15)	0.0045 (14)	−0.0020 (15)
C2	0.039 (2)	0.043 (2)	0.055 (2)	−0.0017 (17)	0.0025 (17)	−0.0056 (18)
C3	0.050 (2)	0.040 (2)	0.078 (3)	−0.0044 (19)	0.010 (2)	0.006 (2)
C4	0.063 (3)	0.051 (3)	0.059 (3)	0.002 (2)	0.009 (2)	0.018 (2)
C5	0.053 (2)	0.052 (2)	0.041 (2)	0.001 (2)	0.0006 (18)	0.0082 (18)
C6	0.0314 (18)	0.041 (2)	0.0354 (18)	0.0056 (15)	0.0039 (14)	0.0009 (15)
C7	0.045 (2)	0.041 (2)	0.0347 (19)	0.0033 (16)	−0.0020 (16)	−0.0043 (16)
C8	0.0409 (19)	0.039 (2)	0.0356 (18)	0.0035 (16)	0.0010 (15)	−0.0034 (15)
C9	0.0349 (18)	0.040 (2)	0.0346 (18)	0.0041 (15)	0.0049 (14)	−0.0041 (15)
C10	0.042 (2)	0.055 (2)	0.037 (2)	−0.0001 (18)	0.0021 (16)	−0.0071 (18)
C11	0.052 (2)	0.064 (3)	0.055 (3)	−0.011 (2)	0.000 (2)	−0.016 (2)
C12	0.052 (2)	0.041 (2)	0.064 (3)	−0.0074 (19)	0.009 (2)	−0.012 (2)
C13	0.061 (3)	0.038 (2)	0.055 (2)	−0.0033 (19)	0.004 (2)	0.0006 (19)
O1	0.081 (2)	0.0545 (19)	0.0429 (16)	0.0025 (16)	−0.0182 (15)	−0.0078 (14)
O2	0.0594 (17)	0.0378 (15)	0.0365 (14)	0.0017 (12)	−0.0069 (12)	−0.0001 (11)
O3	0.074 (2)	0.076 (2)	0.0433 (17)	−0.0031 (18)	−0.0168 (15)	0.0024 (16)

### Geometric parameters (Å, º)

**Table:**

C1—C2	1.393 (5)	C8—C9	1.349 (5)
C1—C6	1.406 (5)	C8—O2	1.360 (4)
C1—C7	1.454 (5)	C8—C13	1.486 (5)
C2—C3	1.372 (6)	C9—C10	1.484 (5)
C2—H2	0.9300	C10—O3	1.222 (5)
C3—C4	1.376 (6)	C10—C11	1.506 (6)
C3—H3	0.9300	C11—C12	1.499 (6)
C4—C5	1.379 (6)	C11—H11A	0.9700
C4—H4	0.9300	C11—H11B	0.9700
C5—C6	1.403 (5)	C12—C13	1.519 (6)
C5—H5	0.9300	C12—H12A	0.9700
C6—C9	1.463 (5)	C12—H12B	0.9700
C7—O1	1.201 (4)	C13—H13A	0.9700
C7—O2	1.387 (5)	C13—H13B	0.9700
			
C2—C1—C6	121.2 (3)	C8—C9—C10	117.4 (3)
C2—C1—C7	118.3 (3)	C6—C9—C10	123.5 (3)
C6—C1—C7	120.6 (3)	O3—C10—C9	122.4 (4)
C3—C2—C1	120.1 (4)	O3—C10—C11	119.6 (4)
C3—C2—H2	120.0	C9—C10—C11	117.9 (4)
C1—C2—H2	120.0	C12—C11—C10	114.8 (3)
C2—C3—C4	119.7 (4)	C12—C11—H11A	108.6
C2—C3—H3	120.2	C10—C11—H11A	108.6
C4—C3—H3	120.2	C12—C11—H11B	108.6
C3—C4—C5	121.2 (4)	C10—C11—H11B	108.6
C3—C4—H4	119.4	H11A—C11—H11B	107.5
C5—C4—H4	119.4	C11—C12—C13	110.5 (4)
C4—C5—C6	120.6 (4)	C11—C12—H12A	109.6
C4—C5—H5	119.7	C13—C12—H12A	109.6
C6—C5—H5	119.7	C11—C12—H12B	109.6
C5—C6—C1	117.2 (4)	C13—C12—H12B	109.6
C5—C6—C9	124.6 (3)	H12A—C12—H12B	108.1
C1—C6—C9	118.1 (3)	C8—C13—C12	110.0 (3)
O1—C7—O2	115.9 (3)	C8—C13—H13A	109.7
O1—C7—C1	127.2 (4)	C12—C13—H13A	109.7
O2—C7—C1	116.9 (3)	C8—C13—H13B	109.7
C9—C8—O2	122.5 (3)	C12—C13—H13B	109.7
C9—C8—C13	126.5 (3)	H13A—C13—H13B	108.2
O2—C8—C13	111.0 (3)	C8—O2—C7	122.7 (3)
C8—C9—C6	119.1 (3)		
			
C6—C1—C2—C3	0.0 (6)	C5—C6—C9—C8	−175.2 (4)
C7—C1—C2—C3	−179.7 (4)	C1—C6—C9—C8	3.7 (5)
C1—C2—C3—C4	1.1 (6)	C5—C6—C9—C10	7.6 (6)
C2—C3—C4—C5	−1.1 (7)	C1—C6—C9—C10	−173.5 (3)
C3—C4—C5—C6	−0.1 (7)	C8—C9—C10—O3	−167.7 (4)
C4—C5—C6—C1	1.2 (6)	C6—C9—C10—O3	9.5 (6)
C4—C5—C6—C9	−179.8 (4)	C8—C9—C10—C11	8.5 (5)
C2—C1—C6—C5	−1.1 (5)	C6—C9—C10—C11	−174.2 (3)
C7—C1—C6—C5	178.5 (3)	O3—C10—C11—C12	−162.4 (4)
C2—C1—C6—C9	179.8 (3)	C9—C10—C11—C12	21.2 (6)
C7—C1—C6—C9	−0.5 (5)	C10—C11—C12—C13	−51.1 (5)
C2—C1—C7—O1	−1.2 (6)	C9—C8—C13—C12	−23.3 (6)
C6—C1—C7—O1	179.1 (4)	O2—C8—C13—C12	156.4 (3)
C2—C1—C7—O2	177.4 (3)	C11—C12—C13—C8	50.7 (5)
C6—C1—C7—O2	−2.3 (5)	C9—C8—O2—C7	1.1 (6)
O2—C8—C9—C6	−4.1 (5)	C13—C8—O2—C7	−178.6 (3)
C13—C8—C9—C6	175.5 (4)	O1—C7—O2—C8	−179.1 (4)
O2—C8—C9—C10	173.2 (3)	C1—C7—O2—C8	2.2 (5)
C13—C8—C9—C10	−7.1 (6)		

### Hydrogen-bond geometry (Å, º)

Cg is the centroid of the C1–C6 benzene ring.

**Table:**

*D*—H···*A*	*D*—H	H···*A*	*D*···*A*	*D*—H···*A*
C11—H11*B*···*Cg*^i^	0.97	2.91	3.723 (5)	142

Symmetry code: (i) −*x*+1, −*y*+1, −*z*+1.
